# Multimorbidity Prevalence, Patterns, and Associated Factors: A Cross-Sectional Study Among Adults Visiting the Family Medicine Department at Hatta Hospital, United Arab Emirates

**DOI:** 10.7759/cureus.100452

**Published:** 2025-12-30

**Authors:** Ayesha Rashid, Muhammad Ismail, Issam Merghani Ahmed Saeed, Fatma Ali Mohamed Saeed

**Affiliations:** 1 Family Medicine, Hatta Hospital, Dubai Health, Dubai, ARE

**Keywords:** chronic diseases, dubai, hatta, multimorbidity, non-communicable diseases, primary care, risk factors, united arab emirates

## Abstract

Background

Multimorbidity, the coexistence of two or more chronic conditions, is an increasing global public health challenge. Although its prevalence is rising internationally, evidence from the United Arab Emirates (UAE) remains limited, particularly in primary care settings serving remote or underserved populations.

Objective

To determine the prevalence, patterns, and associated factors of multimorbidity among adults attending the outpatient Family Medicine Department at Hatta Hospital, Dubai, UAE.

Methods

This cross-sectional study was conducted at the Family Medicine Department of Hatta Hospital, a primary care facility serving the rural Hatta region of Dubai, UAE, from April to July 2025. A total of 262 adults aged ≥18 years were consecutively enrolled. Multimorbidity was defined as the presence of ≥2 chronic conditions according to WHO criteria. Sociodemographic characteristics, lifestyle factors, chronic disease status, and mental health indicators (Patient Health Questionnaire-9 (PHQ-9) and Generalized Anxiety Disorder-7 (GAD-7)) were collected through structured interviews, electronic medical record review, and clinical assessments. Multivariable binary logistic regression was used to identify factors associated with multimorbidity.

Results

The mean age of participants was 45.3±18.5 years; 87 (33.2%) were male, and 175 (66.8%) were female. The prevalence of multimorbidity was 107 (40.8%). Among all participants, 77 (29.4%) had two to four conditions, 12 (4.6%) had five to six conditions, and 18 (6.9%) had more than six conditions. The most common chronic conditions were obesity in 89 (34.0%), hypertension in 80 (30.5%), and diabetes in 73 (27.9%). In multivariable analysis, age and educational level emerged as associated factors. Compared with individuals aged ≥60 years, those aged 18-34 years (adjusted OR=0.03, 95% CI: 0.00-0.21, p=0.001) and 35-59 years (adjusted OR=0.13, 95% CI: 0.03-0.53, p=0.005) had significantly lower odds of multimorbidity. Illiterate individuals (adjusted OR=5.72, 95% CI: 1.32-24.78, p=0.020) and those with primary education (adjusted OR=15.46, 95% CI: 3.07-77.68, p<0.001) had significantly higher odds compared with university graduates.

Conclusion

Multimorbidity is highly prevalent among primary care patients in Hatta, with age and low educational attainment emerging as key factors independently associated with multimorbidity. The predominance of cardiometabolic conditions and their clustering patterns highlight the need for integrated chronic disease management and targeted health literacy interventions, especially for older adults with limited formal education.

## Introduction

Multimorbidity, defined as the coexistence of two or more chronic health conditions in a single individual, has emerged as a pressing global public health challenge. Its prevalence is increasing due to population aging, improved survival from chronic illnesses, and widespread lifestyle transitions associated with urbanization and economic development. Multimorbidity is linked to poor quality of life, higher healthcare utilization, longer hospital stays, and increased mortality, placing a substantial burden on healthcare systems worldwide [1,2].

While substantial research exists in high-income countries, evidence from developing regions, including the Middle East, remains limited. Rapid economic growth in this region has shifted disease patterns toward non-communicable, lifestyle-related conditions such as obesity, hypertension, diabetes, and cardiovascular disease [3]. In the United Arab Emirates (UAE), these chronic diseases are increasingly prevalent, reflecting a well-recognized epidemiological transition [4,5]. However, most available studies originate from major urban centres, and there is minimal research describing the burden of multimorbidity in rural or geographically distinct communities.

Hatta, a mountainous town in eastern Dubai, represents an underserved population with unique sociodemographic, lifestyle, and healthcare access characteristics compared to urban centres. Despite these differences, the prevalence and determinants of multimorbidity in rural UAE settings remain largely unknown [6]. Addressing this knowledge gap is crucial, as rural communities may experience different risk profiles and healthcare needs.

Primary care plays a pivotal role in the early identification and management of multimorbidity by providing continuous, coordinated care. Understanding the burden of multimorbidity and its associated factors in a rural population is essential to inform targeted prevention strategies, optimize service delivery, and guide resource allocation at the community level [3].

Given the UAE's rapid demographic and lifestyle transitions, multimorbidity is expected to rise. However, evidence from rural primary care settings is scarce. Therefore, this study aims to determine the prevalence of multimorbidity and identify associated factors among adults attending the Family Medicine Department at Hatta Hospital. The findings will generate context-specific evidence to support health system planning and improve chronic disease management in rural UAE communities.

## Materials and methods

Study design and setting

This cross-sectional study was conducted in the outpatient Family Medicine Department at Hatta Hospital, Dubai, UAE, from April to July 2025. Ethical approval was obtained from the Mohammed Bin Rashid University of Medicine and Health Sciences Institutional Review Board (MBRU IRB approval number: MBRU IRB-2024-710) and the Dubai Scientific Research Ethics Committee (DSREC reference number: DSREC-04/2025_17) prior to study initiation.

Study population

Adults aged ≥18 years who attended the outpatient primary care clinic for medical consultation during the study period were consecutively enrolled, regardless of nationality. Patients presenting for non-medical or administrative purposes - such as health certificate issuance or occupational assessment, pregnant women, and patients receiving palliative care - were excluded.

Ethical considerations

Given the minimal-risk nature of the study, DSREC (Reference number: DSREC-04/2025_17) approved the use of verbal informed consent. Participants were informed about the study purpose, voluntary participation, confidentiality safeguards, and their right to withdraw at any time. The verbal consent process was documented in each participant's medical record. Confidentiality was ensured through coded identifiers, and all data were stored in password-protected electronic files accessible only to the research team. No imputation was performed for missing data; all variables included in the analysis had complete information.

Sample size calculation

The sample size was calculated using the OpenEpi online calculator, based on a previously reported multimorbidity prevalence of 21.8% in Qatar [7]. Assuming a 95% confidence interval and a 5% margin of error, the minimum required sample size was 262 participants.

Data collection

Data were collected through structured, face-to-face interviews conducted by attending physicians using a standardized proforma. Sociodemographic variables included age, gender, nationality, educational level (illiterate, primary, secondary, college, graduation and above), marital status (single, married, divorced/widowed), residence (urban or rural), employment status (employed, unemployed), and monthly household income in AED (<10,000; 10,000-20,000; 21,000-40,000; 41,000-60,000; >60,000).

Lifestyle-related information included physical activity frequency (daily, three to five times per week, once per week, rarely, never), dietary patterns (healthy, unhealthy), healthcare prioritization (high, medium, low), family support (yes, no), and financial difficulty in accessing nutritious food (yes, no).

Clinical information was obtained through participant self-report of physician-diagnosed chronic illnesses. All reported conditions were systematically verified using electronic medical records, medication history, and relevant laboratory or clinical findings before being documented in the dataset. After confirmation of evidence of existing conditions through these sources, the presence of each condition was documented as positive in the dataset. Chronic conditions assessed included hypertension, diabetes mellitus, dyslipidaemia, ischemic heart disease (IHD), chronic kidney disease (CKD), stroke, asthma, chronic obstructive pulmonary disease (COPD), obesity, osteoarthritis, active cancer, depression, and anxiety. For cancer, only patients with active malignancy (currently undergoing treatment or with documented active disease) were included. Patients with past cancer history who were in complete remission or cured were not classified as having current cancer, maintaining consistency with our assessment of other conditions as current health status.

Definitions and assessment tools

Multimorbidity was defined according to the World Health Organization criteria as the presence of ≥2 chronic conditions in an individual.

Depression was assessed using the Patient Health Questionnaire-9 (PHQ-9), with scores ≥10 (10 or above) indicating probable depression [8]. Anxiety was evaluated using the Generalized Anxiety Disorder-7 (GAD-7), with scores ≥5 (5 or above) indicating probable anxiety. The ≥5 threshold was used to maximize sensitivity for anxiety detection in primary care screening, where early identification of mild-to-moderate symptoms is clinically relevant for early intervention. This lower threshold, compared to the ≥10 cut-off typically used for moderate-to-severe anxiety, captures a broader spectrum of anxiety symptoms appropriate for primary care settings [9].

Obesity was classified using the WHO body mass index (BMI) categories, with height and weight measured using standard calibrated equipment during clinic visits [10].

A healthy diet was defined as regular consumption of vegetables, fruits, lean proteins, and low-fat foods, whereas an unhealthy diet reflected frequent intake of processed foods, bakery items, fast foods, or carbonated beverages. Family support was defined as the presence of emotional, physical, or financial assistance during ongoing treatment.

The priority of accessing healthcare services was evaluated by asking participants to rate on a scale of 1-10 how much priority they gave to accessing healthcare services during the past six months. Scores were categorized as: high priority (≥8), medium priority (5-7), and low priority (<5). This variable assessed participants' self-reported prioritization of healthcare-seeking behaviour. The study proforma included sociodemographic variables, such as age, gender, education, residence, monthly income, marital status, employment status, physical activity, diet, priority of accessing healthcare services, family support, and financial difficulty in accessing a nutritional diet. Clinical variables included coexisting diseases such as hypertension, diabetes, dyslipidaemia, IHD, depression, anxiety, cancer, etc.

Statistical analysis

Data were entered into Microsoft Excel (Microsoft® Corp., Redmond, WA) and analysed using Statistical Product and Service Solutions (SPSS, version 27.0; IBM SPSS Statistics for Windows, Armonk, NY). Continuous variables were summarized as means ± standard deviations, and categorical variables as frequencies and percentages.

Binary logistic regression was applied to determine the association of patients' features with multimorbidity. First, bivariate odds ratios with their 95% confidence intervals were computed. Variables with p<0.25 were entered in a final regression model to compute adjusted odds ratios. Before fitting the multivariable regression model, the assumption of multicollinearity was assessed with a correlation matrix and variance inflation factors (VIF>10). P-value ≤0.05 was deemed statistically significant in the final regression model.

## Results

Baseline characteristics

A total of 262 participants were enrolled, with a mean age of 45.3±18.5 years (range: 18-96 years). Of these, 87 (33.2%) were male, 175 (66.8%) were female, 216 (82.4%) were Emirati nationals, and 204 (77.9%) resided in rural areas. More than three-quarters (204, 77.9%) were married, divorced, or widowed. More than half reported an unhealthy diet (161, 61.5%) and lack of family support (150, 57.3%). Additionally, 107 participants (40.8%) reported financial difficulties in accessing nutritious food (Table 1).

**Table 1:** Baseline characteristics AED: United Arab Emirates Dirham

Variables	Groups	Frequency	Percentage
Age	18-34 years	85	32.4
	35-59 years	116	44.3
	≥60 years	61	23.3
Gender	Male	87	33.2
	Female	175	66.8
Residence	Rural	204	77.9
	Urban	58	22.1
Ethnicity	Asian	15	5.7
	Saudi	1	0.4
	Omani	20	7.6
	Emirati	216	82.4
	Others	10	3.8
Education	Illiterate	73	27.9
	Primary	33	12.6
	Secondary	73	27.9
	College	38	14.5
	Graduation and above	45	17.2
Marital status	Single	58	22.1
	Married	177	67.6
	Divorced/widowed	27	10.3
Employment status	Employed	107	40.8
	Unemployed	155	59.2
Monthly income in AED	<10k	74	28.2
	10k-20k	70	26.7
	21k-40k	26	9.9
	41k-60k	38	14.5
	>60k	54	20.6
Physical activity	Daily	36	13.7
	3-5 times per week	63	24
	Once a week	36	13.7
	Rarely	104	39.7
	Never	23	8.8
Diet	Healthy	101	38.5
	Unhealthy	161	61.5
Healthcare prioritization (1-10 scale)	High	125	47.7
	Medium	104	39.7
	Low	33	12.6
Family support	Yes	112	42.7
	No	150	57.3
Financial difficulty in accessing nutritious food	Yes	107	40.8
	No	155	59.2

Prevalence and distribution of multimorbidity

**Figure 1:**
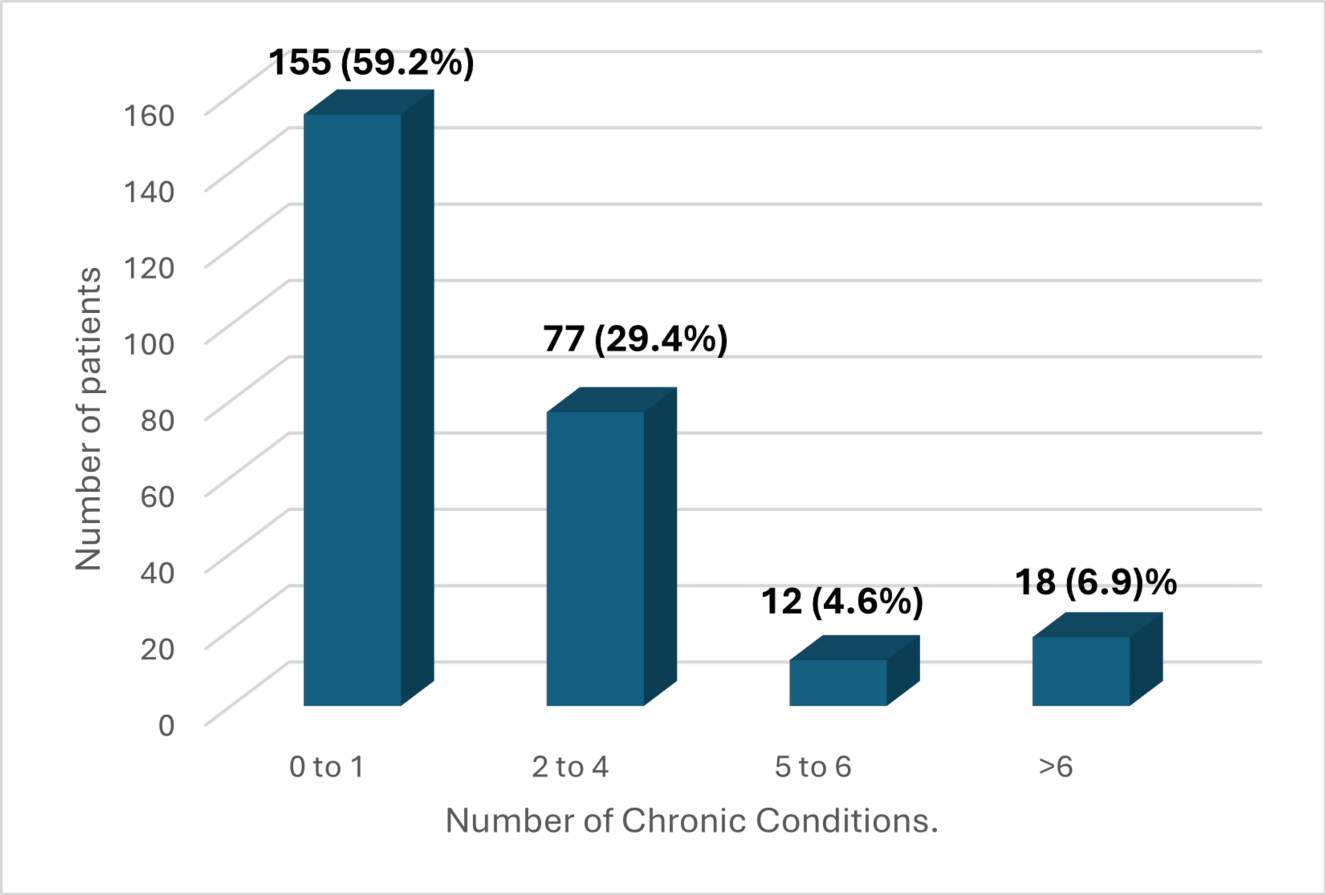
Distribution of chronic condition burden among study participants (N=262) Among all participants, 59.2% had 0-1 chronic conditions (no multimorbidity), while 40.8% had multimorbidity (≥2 conditions). The median number of chronic conditions was 1 (range: 0-11).

The overall prevalence of multimorbidity (≥2 chronic conditions) was 107 (40.8%), while 155 (59.2%) had 0-1 chronic condition. Among all participants, 77 (29.4%) had two to four conditions, 12 (4.6%) had five to six conditions, and 18 (6.9%) had more than six conditions. The median number of chronic conditions in the overall sample was 1 (range: 0-11) (Figure 1).

Prevalence of individual chronic conditions

The most prevalent chronic condition was obesity in 89 (34.0%), followed by hypertension in 80 (30.5%) and diabetes mellitus in 73 participants (27.9%). Other conditions included ischemic heart disease in 63 (24.0%), cancer in five (1.9%), dyslipidaemia in 36 (13.7%), chronic kidney disease in 34 (13.0%), anxiety in 30 (11.5%), stroke in 24 (9.2%), osteoarthritis in 22 (8.4%), asthma in 21 (8.0%), depression in 13 (5.0%), and chronic obstructive pulmonary disease in 11 participants (4.2%).

Chronic conditions by multimorbidity status

Table 2 presents the distribution of each chronic condition stratified by multimorbidity status. Several conditions occurred exclusively among participants with multimorbidity: all 11 patients with chronic obstructive pulmonary disease (100%), all 34 with chronic kidney disease (100%), all 24 with stroke (100%), and all 13 with depression (100%). Additionally, multimorbidity was highly prevalent among patients with hypertension (77 of 80, 96.3%), cancer (5 of 5, 100%), osteoarthritis (21 of 22, 95.5%), and ischemic heart disease (58 of 63, 92.1%). In contrast, obesity showed a near-equal distribution between isolated and multimorbid presentations (44 vs 45, 49.4% vs 50.6%).

**Table 2:** Frequency distribution of chronic conditions stratified by multimorbidity status COPD: Chronic obstructive pulmonary disease; IHD: Ischemic heart disease; CKD: Chronic kidney disease

Morbidities	Single condition n (%)	≥2 conditions n (%)	Total n (%)
Obesity	44 (49.4)	45 (50.6)	89 (34.0)
Dyslipidemia	13 (36.1)	23 (63.9)	36 (13.7)
Hypertension	3 (3.8)	77 (96.3)	80 (30.5)
Diabetes	11 (15.1)	62 (84.9)	73 (27.9)
Asthma	3 (14.3)	18 (85.7)	21 (8.0)
COPD	0 (0.0)	11 (100.0)	11 (4.2)
IHD	5 (7.9)	58 (92.1)	63 (24.0)
CKD	0 (0.0)	34 (100.0)	34 (13.0)
Osteoarthritis	1 (4.5)	21 (95.5%)	22 (8.4)
Cancer	0 (0.0)	5 (100.0)	5 (1.9)
Depression	0 (0.0)	13 (100.0)	13 (5.0)
Anxiety	5 (16.7)	25 (83.3)	30 (11.5)
Stroke	0 (0.0)	24 (100.0)	24 (9.2)

Bivariate analysis of factors associated with multimorbidity

Bivariate logistic regression was performed to examine the association between each patient characteristic and multimorbidity (Table 3). This analysis revealed a strong inverse relationship between age and multimorbidity. Compared with participants aged ≥60 years, individuals aged 18-34 years had significantly lower odds (OR=0.01, 95% CI: 0.01-0.02, p<0.001), as did those aged 35-59 years (OR=0.04, 95% CI: 0.02-0.10, p<0.001). Educational level demonstrated a marked gradient: compared with university graduates, illiterate participants had substantially higher odds of multimorbidity (OR=12.48, 95% CI: 4.65-33.45, p<0.001), as did those with primary education (OR=29.25, 95% CI: 8.52-100.41, p<0.001). Being married, divorced, or widowed was associated with higher odds compared with being single (OR=10.60, 95% CI: 4.07-27.60, p<0.001). Employment was protective (OR=0.34, 95% CI: 0.20-0.58, p<0.001). Lower household income, particularly <10,000 AED, showed significantly higher odds (OR=9.75, 95% CI: 4.19-22.71, p<0.001). Rural residence was associated with lower odds (OR=0.47, 95% CI: 0.26-0.85, p=0.013). Regarding healthcare prioritization, participants with medium priority (score 5-7 on 1-10 scale) had significantly higher odds of multimorbidity compared to those with low priority (OR=5.26, 95% CI: 2.21-12.51, p<0.001), while those with high priority (score ≥8) showed no significant association (OR=0.81, 95% CI: 0.34-1.92, p=0.627). This pattern likely reflects reverse causation, where individuals with multiple chronic conditions place moderate priority on healthcare while managing competing life demands. Protective associations were also observed for family support and the absence of financial difficulties in accessing nutritious food. Gender, ethnicity, and physical activity were not significantly associated with multimorbidity.

**Table 3:** Bivariate logistic regression analysis of factors associated with multimorbidity OR: Odds ratio; CI: Confidence interval; AED: United Arab Emirates Dirham; *Significant at p<0.05

Variables	Groups	Multimorbidity		OR (95% CI)	p-value
		Yes n(%)	No n(%)		
Age	18-34 years	6 (7.1)	79 (92.9)	0.01 (0.01-0.02)	*<0.001
	35-59 years	44 (37.9)	72 (62.1)	0.04 (0.02-0.10)	*<0.001
	≥60 years	57 (93.4)	4 (6.6)	Reference category	
Gender	Male	36 (41.4)	51 (58.6)	1.03 (0.61-1.74)	0.9
	Female	71 (40.6)	104 (59.4)	Reference category	
Residence	Rural	75 (36.8)	129 (63.2)	0.47 (0.26-0.85)	*0.013
	Urban	32 (55.2)	26 (44.8)	Reference category	
Ethnicity	Asian	6 (37.5)	10 (62.5)	0.90 (0.17-4.54)	0.899
	Omani	11 (55)	9 (45)	1.83 (0.39-8.56)	0.441
	Emirati	86 (39.8)	130 (60.2)	0.99 (0.27-3.62)	0.991
	Others	4 (40)	6 (60)	Reference category	
Education	Illiterate	48 (65.8)	25 (34.2)	12.48 (4.65-33.45)	*<0.001
	Primary	27 (81.8)	6 (18.2)	29.25 (8.52-100.41)	*<0.001
	Secondary	21 (28.8)	52 (71.2)	2.62 (0.96-7.12)	0.058
	College	5 (13.2)	33 (86.8)	0.98 (0.27-3.52)	0.981
	Graduation and above	6 (13.3)	39 (86.7)	Reference category	
Marital status	Single	5 (8.6)	53 (91.4)	Reference category	
	Married/divorced/widowed	102 (50)	102 (50)	10.60 (4.07-27.60)	*<0.001
Employment status	Employed	28 (26.2)	79 (73.8)	0.34 (0.20-0.58)	*<0.001
	Unemployed	79 (51)	76 (49)	Reference category	
Monthly income in AED	<10k	51 (68.9)	23 (31.1)	9.75 (4.19-22.71)	*<0.001
	10k-20k	33 (47.1)	37 (52.9)	3.92 (1.71-9.01)	*0.001
	21k-40k	7 (26.9)	19 (73.1)	1.62 (0.54-4.89)	0.392
	41k-60k	6 (15.8)	32 (84.2)	0.83 (0.27-2.50)	0.734
	>60k	10 (18.5)	44 (81.5)	Reference category	
Physical activity	Daily	15 (41.7)	21 (58.3)	2.02 (0.65-6.34)	0.226
	3-5 times per week	16 (25.4)	47 (74.6)	0.96 (0.32-2.86)	0.948
	Once a week	16 (44.4)	20 (55.6)	2.26 (0.73-7.08)	0.159
	Rarely	54 (51.9)	50 (48.1)	3.06 (1.11-8.37)	*0.030
	Never	6 (26.1)	17 (73.9)	Reference category	
Diet	Healthy	49 (48.5)	52 (51.5)	1.67 (1.01-2.78)	*0.046
	Unhealthy	58 (36)	103 (64)	Reference category	
Healthcare prioritization (1-10 scale)	High	29 (23.2)	96 (76.8)	0.81 (0.34-1.92)	0.627
	Medium	69 (66.3)	35 (33.7)	5.26 (2.21-12.51)	*<0.001
	Low	9 (27.3)	24 (72.7)	Reference category	
Family support	Yes	35 (31.3)	77 (68.8)	0.49 (0.29-0.82)	*0.007
	No	72 (48)	78 (52)	Reference category	
Financial difficulty in accessing nutritious food	Yes	71 (66.4)	36 (33.6)	6.52 (3.77-11.27)	*<0.001
	No	36 (23.2)	119 (76.8)	Reference category	

Multivariable analysis of associated factors

In multivariable logistic regression (Table 4), only age and educational level remained statistically significant associated factors of multimorbidity. Compared with adults aged ≥60 years, younger participants had substantially lower adjusted odds: 18-34 years (aOR=0.03, 95% CI: 0.00-0.21, p=0.001) and 35-59 years (aOR=0.13, 95% CI: 0.03-0.53, p=0.005). Lower educational attainment remained strongly associated with multimorbidity: compared with university graduates, illiterate participants had nearly six-fold higher odds (aOR=5.72, 95% CI: 1.32-24.78, p=0.020), while those with only primary education had fifteen-fold higher odds (aOR=15.46, 95% CI: 3.07-77.68, p<0.001). After adjustment, marital status, residence, employment, household income, physical activity, diet, healthcare access, family support, and financial difficulty in accessing nutritious food no longer retained statistical significance.

**Table 4:** Multivariable logistic regression analysis of factors associated with multimorbidity aOR: Adjusted odds ratio; CI: Confidence interval; *Significant at p<0.05

Variables	Groups	aOR	95% CI		p-value
			Lower	Upper	
Age	18-34 years	0.03	0	0.21	*0.001
	35-59 years	0.13	0.03	0.53	*0.005
	≥60 years	Reference category			
Residence	Rural	0.54	0.17	1.61	0.27
	Urban	Reference category			
Education	Illiterate	5.72	1.32	24.78	*0.020
	Primary	15.46	3.07	77.68	*<0.001
	Secondary	1.31	0.38	4.53	0.66
	College	0.7	0.12	4.01	0.69
	Graduation or above	Reference category			
Marital status	Single	Reference category			
	Married/divorced/widowed	1.69	0.35	4.53	0.515
Employment status	Employed	1.59	0.58	4.37	0.367
	Unemployed	Reference category			
Monthly income in AED	<10k	4.17	0.79	21.82	0.09
	10k-20k	1.76	0.34	9.14	0.499
	21k-40k	1	0.15	6.58	0.997
	41k-60k	1.16	0.24	5.61	0.847
	>60k	Reference category			
Physical activity	Daily	0.64	0.09	4.56	0.54
	3-5 times weekly	1.27	0.22	7.44	0.782
	Once a week	3.56	0.55	8.98	0.147
	Rarely	2.59	0.47	5.11	0.326
	Never	Reference category			
Diet	Healthy	1.14	0.48	2.71	0.758
	Unhealthy	Reference category			
Healthcare prioritization (1-10 scale)	High	1.03	0.23	4.59	0.966
	Medium	0.64	0.12	3.3	0.602
	Low	Reference category			
Family support	Yes	0.42	0.13	1.34	0.143
	No	Reference category			
Financial difficulty in accessing nutritious food	Yes	1.95	0.32	6.23	0.472
	No	Reference category			

Sensitivity analysis

A sensitivity analysis was conducted to examine clustering patterns by grouping chronic conditions into cardiometabolic versus non-cardiometabolic categories. Among the 107 participants with multimorbidity, cardiometabolic conditions (obesity, diabetes, hypertension, dyslipidaemia, ischemic heart disease, stroke) showed high co-occurrence, with most individuals having two or more cardiometabolic conditions simultaneously. Detailed breakdown of conditions by category is provided in Tables 5-6.

**Table 5:** Cardiometabolic conditions (N=262) IHD: Ischemic heart disease

Cardiometabolic Conditions	Frequency (%)
Obesity	89 (34.0)
Dyslipidemia	36 (13.7)
Hypertension	80 (30.5)
Diabetes	73 (27.9)
IHD	63 (24.0)
Stroke	24 (9.2)

**Table 6:** Non-cardiometabolic conditions (N=262) COPD: Chronic obstructive pulmonary disease; CKD: Chronic kidney disease

Non-communicable diseases	Frequency (%)
Chronic respiratory diseases	
Asthma	21 (8.0)
COPD	11 (4.2)
Mental Health	
Depression	13 (5.0)
Anxiety	30 (11.5)
Musculoskeletal disorders	
Osteoarthritis	22 (8.4)
Renal Diseases	
CKD	34 (13.0)
Cancer	
Active cancer	5 (1.9)

## Discussion

In this study, the clinic-based prevalence of multimorbidity (≥2 chronic conditions) among adults attending primary care clinics at Hatta Hospital, UAE, was 107 (40.8%), with an additional 77 (29.4%) having a single chronic condition. This estimate aligns closely with the pooled global prevalence of 37.2% reported in a recent meta-analysis of community-based studies [11]. Regional estimates vary considerably: Pakistan reported 47% among low-income urban adults with cardiometabolic disease [12]; Iran, 36.6% [13]; India, 40.2% [14]; and Brazil, 57% [15]. In Saudi Arabia, estimates ranged from 7.1% to 77.6%, depending on study design, population characteristics, and definitions used [16]. Such variability likely reflects differences in target populations (community-based versus hospital-based), age structures, data sources (self-report versus clinical records), and socioeconomic contexts. The prevalence observed in our rural Hatta population aligns with the upper range of regional estimates, highlighting rising multimorbidity in Gulf countries, particularly within primary care settings where chronic disease detection is systematic and comprehensive.

Our observed prevalence was substantially higher than the 21.8% reported in the Qatar study used for our sample size calculation [7]. Several factors may explain this discrepancy. First, our population was drawn from a rural mountainous community, potentially differing in risk profiles from urban Qatari populations. Second, systematic screening for depression and anxiety using validated tools (PHQ-9 and GAD-7) likely increased detection of mental health conditions, which are frequently underdiagnosed in routine primary care. Third, our comprehensive data collection - including self-report, electronic medical records, medication reviews, and clinical assessments - may have improved chronic condition ascertainment compared with studies relying solely on self-report. Fourth, the rising prevalence of non-communicable diseases in the UAE over recent years may have contributed to higher rates than previously reported. Across the UAE, rapid urbanization, demographic shifts, and lifestyle modifications have contributed to growing multimorbidity burden, prompting healthcare systems to enhance chronic disease management programs. These findings underscore the importance of context-specific research for effective health service planning.

The most prevalent conditions were obesity in 89 (34.0%), hypertension in 80 (30.5%), and diabetes in 73 (27.9%), reflecting the well-documented cardiometabolic disease epidemic in Gulf countries. Most chronic conditions rarely occurred in isolation: 77 of 80 hypertensive patients (96.3%) had at least one additional condition, and all participants with chronic obstructive pulmonary disease, chronic kidney disease, depression, or stroke had multimorbidity. This clustering, particularly of cardiometabolic risk factors, highlights the interconnected nature of chronic disease pathways and supports the need for integrated care approaches rather than disease-specific programs.

Age was strongly associated with multimorbidity. Multimorbidity was the lowest among participants aged 18-34 years (6 of 85, 7.1%), increased in those aged 35-59 years (44 of 116, 37.9%), and was highest among individuals ≥60 years (57 of 61, 93.4%). After adjustment, younger age groups had significantly lower odds of multimorbidity compared to those ≥60 years (aOR=0.03 for 18-34 years; aOR=0.13 for 35-59 years). Similar age-related trends have been reported in other populations: in a large urban South Asian cohort (Delhi, Chennai, Karachi), multimorbidity prevalence increased from 9.4% overall to 37% among individuals aged ≥60 years [17]. In Brazil, a national population-based study reported that individuals aged >60 years were almost 20 times more likely to have multimorbidity compared to those aged 18-29 years [18]. Educational level also emerged as a strong associated factor, with illiterate individuals and those with only primary education having significantly higher adjusted odds than university graduates (aOR=5.72 and aOR=15.46, respectively). However, the wide confidence intervals for these estimates (particularly for primary education: 3.07-77.68) reflect the limited sample size and should be interpreted with appropriate caution. A systematic review of multimorbidity in Southeast Asia found that educational level presented three different scenarios to the odds of multimorbidity in the studies included: educational level sometimes reduced odds of multimorbidity; sometimes increased odds; and in some studies, there was no association whatsoever [19]. Other studies have shown that educational level acted as a fundamental socioeconomic determinant of multimorbidity, operating through a number of mechanisms, including occupation, income, health literacy, and access to healthcare; however, the association in each of these studies was contingent on setting, age of the population, or disease profile [20, 21]. Our findings align with studies showing strong protective effects of higher education, which may reflect better health literacy, greater access to healthcare resources, and healthier lifestyle choices among more educated individuals in our setting.

Other socioeconomic variables, including income, marital status, and residence, showed associations in bivariate analysis but were attenuated in multivariable models, suggesting confounding effects. In our study, rural residence was associated with lower odds of multimorbidity in bivariate analysis (OR=0.47, 95% CI: 0.26-0.85, p=0.013), but this association did not persist after adjustment. The literature on rural-urban differences in multimorbidity risk is mixed: a meta-analysis indicated that urban residency could lead to a greater likelihood of multimorbidity compared to rural residency [22], while another systematic review focused on area-level and household determinants indicated that the most deprived areas had the highest likelihood (pooled OR=1.42, 95% CI: 1.41-1.42) [23]. These inconsistent findings likely reflect the complex interplay between urbanization, socioeconomic deprivation, and healthcare access across different contexts. Gender, physical activity, diet, and family support were not independently associated with multimorbidity in multivariable analysis. Both a healthy diet and sufficient physical activity measures are independently and jointly associated with a decreased risk and severity of multimorbidity as reported in the literature [24,25]. Similarly, family support did not show an independent association in our adjusted model, although a large nationwide Swedish family study investigated aggregations of multimorbidity within families that imply shared genetic and family lifestyle contributions to multimorbidity risk, but did not directly investigate family support as a psychosocial factor [26]. The lack of significant associations in our adjusted models may reflect measurement limitations inherent in self-reported data, the cross-sectional study design, which cannot capture temporal relationships, or the possibility that these factors operate through the stronger factors we identified (age and education). The diet finding showed a counterintuitive direction in bivariate analysis (OR=1.67 for healthy diet), likely reflecting reverse causation where individuals with chronic conditions adopt healthier diets after diagnosis.

Healthcare prioritization showed a complex pattern in bivariate analysis. Participants who rated medium priority for accessing healthcare services (score 5-7 on a 1-10 scale) had significantly higher odds of multimorbidity compared to those with low priority (OR=5.26, 95% CI: 2.21-12.51, p<0.001), while those with high priority (score ≥8) showed no significant association (OR=0.81, 95% CI: 0.34-1.92, p=0.627). This pattern likely reflects reverse causation inherent in cross-sectional studies: individuals with multiple chronic conditions place moderate - rather than extreme - priority on healthcare as they balance health needs with other life demands. However, healthcare prioritization was not significantly associated with multimorbidity in the adjusted model (high priority: aOR=1.03, 95% CI: 0.23-4.59, p=0.966; medium priority: aOR=0.64, 95% CI: 0.12-3.30, p=0.602), suggesting that this relationship was confounded by age and educational factors. International evidence consistently shows that individuals with multimorbidity utilize healthcare services more frequently and face challenges in accessing appropriate, coordinated care [27,28]. While our data cannot directly assess quality of care or continuity, the lack of an independent association between healthcare prioritization and multimorbidity in the adjusted model suggests that access alone may be insufficient. Quality of care, patient engagement, care coordination, and health literacy may play more decisive roles in shaping chronic disease outcomes than mere availability or prioritization of services, though this hypothesis requires further investigation in longitudinal studies.

These findings have important implications for primary care in the UAE. Specifically, the very high multimorbidity prevalence among older adults (93.4% in those ≥60 years) underscores the urgent need for age-friendly service delivery approaches. The strong independent association with low educational attainment (aOR=15.46 for primary education, aOR=5.72 for illiteracy compared to university graduates) highlights the critical need for health literacy interventions specifically targeting populations with limited formal education. The predominant clustering of cardiometabolic conditions (obesity, hypertension, diabetes) emphasizes the need for integrated chronic disease management models that address multiple interconnected conditions simultaneously rather than disease-specific approaches.

Impact of this study

This study provides the first comprehensive evidence on multimorbidity patterns in rural UAE primary care, demonstrating that 93.4% of adults aged ≥60 years have multimorbidity, with strong clustering of cardiometabolic conditions. These findings have three key implications.

First, the high prevalence and cardiometabolic clustering pattern necessitate integrated, patient-centred primary care models that manage multiple chronic conditions simultaneously, moving away from disease-specific approaches. Second, the 15-fold higher odds of multimorbidity among individuals with only primary education (compared to university graduates) identify education as a critical social determinant of health, highlighting the urgent need for health literacy interventions targeting populations with limited formal education. Third, policymakers can use these context-specific findings to guide resource allocation toward age-friendly services and comprehensive chronic disease management programs in rural and semi-urban communities. By providing evidence from an understudied rural population, this study contributes actionable insights to inform healthcare policy and strengthen primary care services aimed at improving outcomes for adults living with multiple chronic conditions.

Strengths and limitations

Strengths

This study has several strengths, including the use of a comprehensive multisource approach to ascertain chronic conditions through self-report, electronic medical records, medication reviews, and clinical assessments. The inclusion of validated mental health screening tools (PHQ-9 and GAD-7) enhances the comprehensiveness of multimorbidity assessment. Additionally, the study addresses an understudied population in rural UAE, providing valuable insights for primary care planning.

Limitations

However, limitations include its cross-sectional design, which prevents causal inferences and may be subject to reverse causation. Conducting the study in a single primary care centre limits generalizability, and findings should not be extrapolated at a community level. Specifically, this study recruited clinic attendees over a four-month period, and the observed prevalence reflects multimorbidity among individuals actively seeking healthcare rather than a community-based sample; thus, estimates may not represent the broader Hatta population. The reliance on self-reported data for certain lifestyle factors may introduce recall bias. Moreover, other variables such as family support, healthcare access, and lifestyle variables were not evaluated with a standardized, validated tool, which may impact findings. We also acknowledge the possibility of overfitting of the regression model because of the limited sample size, which contributes to the wide range of confidence intervals of odds ratios. Additionally, the use of GAD-7 ≥5 rather than the more restrictive ≥10 cut-off may result in higher anxiety prevalence compared to studies using the moderate-to-severe threshold, though this approach was intentionally chosen to maximize sensitivity for screening in primary care. Finally, the sample size, while adequate for prevalence estimation, limits the precision of estimates for subgroup analyses and may have constrained the detection of additional significant associations in multivariable models.

## Conclusions

This study demonstrates a high prevalence of multimorbidity, affecting 107 (40.8%) of adults attending primary care in rural UAE, with cardiometabolic conditions such as obesity, hypertension, and diabetes predominating and frequently clustering. Older age and lower educational attainment were associated with multimorbidity, highlighting vulnerable groups that may be critically evaluated during routine practice. The findings of the present study suggest factors for primary care planning, but the cross-sectional nature of the study limits temporal interpretation. Future longitudinal research is essential to clarify causal pathways, evaluate the effectiveness of integrated care interventions, and explore the role of social and family support in moderating health outcomes. By providing context-specific evidence from a rural UAE population, this study contributes valuable insights to inform healthcare policy, resource allocation, and the design of primary care services aimed at improving outcomes for adults living with multiple chronic conditions.

## References

[1] Smith SM, Wallace E, Clyne B, Boland F, Fortin M (2021). Interventions for improving outcomes in patients with multimorbidity in primary care and community setting: a systematic review. Syst Rev.

[2] Zhou Y, Wang R, Chen S (2023). Interventions and management on multimorbidity: a scoping review. Int J Environ Res Public Health.

[3] Lee S, Kim H (2022). Advancing multimorbidity management in primary care. Int J Environ Res Public Health.

[4] Mezhal F, Oulhaj A, Abdulle A (2023). High prevalence of cardiometabolic risk factors amongst young adults in the United Arab Emirates: the UAE Healthy Future Study. BMC Cardiovasc Disord.

[5] Mate K, Bryan C, Deen N, McCall J (2017). Review of health systems of the Middle East and North Africa region. International Encyclopedia of Public Health, Second Edition.

[6] Singh K, Alomari A, Lenjawi B (2022). Prevalence of multimorbidity in the Middle East: a systematic review of observational studies. Int J Environ Res Public Health.

[7] Mohideen FS, Rajkumar Honest PC, Syed MA, David KV, Abdulmajeed J, Ramireddy N (2021). Prevalence of multimorbidity among adults attending primary health care centres in Qatar: a retrospective cross-sectional study. J Family Med Prim Care.

[8] Manea L, Gilbody S, McMillan D (2012). Optimal cut-off score for diagnosing depression with the patient health questionnaire (PHQ-9): a meta-analysis. CMAJ.

[9] Spitzer RL, Kroenke K, Williams JB, Löwe B (2006). A brief measure for assessing generalized anxiety disorder: the GAD-7. Arch Intern Med.

[10] WHO Expert Consultation (2004). Appropriate body-mass index for Asian populations and its implications for policy and intervention strategies. Lancet.

[11] Chowdhury SR, Chandra Das D, Sunna TC, Beyene J, Hossain A (2023). Global and regional prevalence of multimorbidity in the adult population in community settings: a systematic review and meta-analysis. EClinicalMedicine.

[12] Jawed M, Inam S, Shah N, Shafique K (2020). Association of obesity measures and multimorbidity in Pakistan: findings from the IMPACT study. Public Health.

[13] Aminisani N, Rastgou L, Shamshirgaran SM, Sarbakhsh P, Ghaderi S, Hyde M (2020). Predictors of multimorbidity among the Kurdish population living in the northwest of Iran. BMC Public Health.

[14] Saqib A, Nawab T, Ahmad A, Khalique N (2025). Multimorbidity and its correlates in middle-aged and older adults in a city of North India. J Family Med Prim Care.

[15] Christofoletti M, Duca GF, Umpierre D, Malta DC (2019). Chronic noncommunicable diseases multimorbidity and its association with physical activity and television time in a representative Brazilian population. Cad Saude Publica.

[16] Alnuwaysir N, Alturki H, Almalki B, Bin Amer L, Alotaibi R (2025). Review of multimorbidity in Saudi Arabia: prevalence, gender differences, and common chronic diseases. J Multimorb Comorb.

[17] Singh K, Patel SA, Biswas S (2019). Multimorbidity in South Asian adults: prevalence, risk factors and mortality. J Public Health (Oxf).

[18] Li XL, Huang H, Lu Y, Stafford RS, Lima SM, Mota C, Shi X (2023). Prediction of multimorbidity in Brazil: latest fifth of a century population study. JMIR Public Health Surveill.

[19] Feng X, Kelly M, Sarma H (2021). The association between educational level and multimorbidity among adults in Southeast Asia: a systematic review. PLoS One.

[20] Pathirana TI, Jackson CA (2018). Socioeconomic status and multimorbidity: a systematic review and meta-analysis. Aust N Z J Public Health.

[21] Chen YH, Karimi M, Rutten-van Mölken MP (2020). The disease burden of multimorbidity and its interaction with educational level. PLoS One.

[22] Asogwa OA, Boateng D, Marzà-Florensa A, Peters S, Levitt N, van Olmen J, Klipstein-Grobusch K (2022). Multimorbidity of non-communicable diseases in low-income and middle-income countries: a systematic review and meta-analysis. BMJ Open.

[23] Ingram E, Ledden S, Beardon S (2021). Household and area-level social determinants of multimorbidity: a systematic review. J Epidemiol Community Health.

[24] Delpino FM, de Lima AP, da Silva BG, Nunes BP, Caputo EL, Bielemann RM (2022). Physical activity and multimorbidity among community-dwelling older adults: a systematic review with meta-analysis. Am J Health Promot.

[25] Abbad-Gomez D, Carballo-Casla A, Beridze G (2025). Dietary patterns and accelerated multimorbidity in older adults. Nat Aging.

[26] Zöller B, Pirouzifard M, Holmquist B, Sundquist J, Halling A, Sundquist K (2023). Familial aggregation of multimorbidity in Sweden: national explorative family study. BMJ Med.

[27] Palladino R, Tayu Lee J, Ashworth M, Triassi M, Millett C (2016). Associations between multimorbidity, healthcare utilisation and health status: evidence from 16 European countries. Age Ageing.

[28] Jørgensen CV, Larsen HH, Siersma V, Holm A (2025). The association between multimorbidity and needs-based quality of life in primary care: a cross-sectional questionnaire study. Scand J Prim Health Care.

